# Development of an anti-*Pseudomonas aeruginosa* therapeutic monoclonal antibody WVDC-5244

**DOI:** 10.3389/fcimb.2023.1117844

**Published:** 2023-04-14

**Authors:** Alexander M. Horspool, Emel Sen-Kilic, Aaron C. Malkowski, Scott L. Breslow, Margalida Mateu-Borras, Matthew S. Hudson, Mason A. Nunley, Sean Elliott, Krishanu Ray, Greg A. Snyder, Sarah Jo Miller, Jason Kang, Catherine B. Blackwood, Kelly L. Weaver, William T. Witt, Annalisa B. Huckaby, Gage M. Pyles, Tammy Clark, Saif Al Qatarneh, George K. Lewis, F. Heath Damron, Mariette Barbier

**Affiliations:** ^1^ Department of Microbiology, Immunology, and Cell Biology, West Virginia University, Morgantown, WV, United States; ^2^ Vaccine Development Center, West Virginia University Health Sciences Center, Morgantown, WV, United States; ^3^ University of Maryland, Baltimore School of Medicine, Division of Vaccine Research, Institute of Human Virology, Baltimore, MD, United States; ^4^ Department of Pediatrics, Division of Cystic Fibrosis, West Virginia University, Morgantown, WV, United States

**Keywords:** *Pseudomonas aeruginosa*, monoclonal antibody, immunotherapy, anti-microbial, passive immunization

## Abstract

The rise of antimicrobial-resistant bacterial infections is a crucial health concern in the 21st century. In particular, antibiotic-resistant *Pseudomonas aeruginosa* causes difficult-to-treat infections associated with high morbidity and mortality. Unfortunately, the number of effective therapeutic interventions against antimicrobial-resistant *P. aeruginosa* infections continues to decline. Therefore, discovery and development of alternative treatments are necessary. Here, we present pre-clinical efficacy studies on an anti-*P. aeruginosa* therapeutic monoclonal antibody. Using hybridoma technology, we generated a monoclonal antibody and characterized its binding to *P. aeruginosa in vitro* using ELISA and fluorescence correlation spectroscopy. We also characterized its function *in vitro* and *in vivo* against *P. aeruginosa*. The anti-*P. aeruginosa* antibody (WVDC-5244) bound *P. aeruginosa* clinical strains of various serotypes *in vitro*, even in the presence of alginate exopolysaccharide. In addition, WVDC-5244 induced opsonophagocytic killing of *P. aeruginosa in vitro* in J774.1 murine macrophage, and complement-mediated killing. In a mouse model of acute pneumonia, prophylactic administration of WVDC-5244 resulted in an improvement of clinical disease manifestations and reduction of *P. aeruginosa* burden in the respiratory tract compared to the control groups. This study provides promising pre-clinical efficacy data on a new monoclonal antibody with therapeutic potential for *P. aeruginosa* infections.

## Introduction

*P. aeruginosa* is a major Gram-negative opportunistic pathogen that causes difficult-to-treat infections due to high levels of multi-drug resistance (MDR). In 2017, MDR *P. aeruginosa* infections were responsible for 32,600 cases and 2,700 deaths in the U.S. alone, and were associated with over $767 million USD of healthcare costs (Centers for Disease Control and Prevention, 2019). *P. aeruginosa* is responsible for 7.1 to 7.3% of all healthcare-associated infections, and is the most common etiological agent of nosocomial pneumonia (Magill et al., 2014; Weiner et al., 2016). *P. aeruginosa* is also responsible for urinary tract, surgical site, burn wound, and bloodstream infections (Estahbanati et al., 2002; Motbainor et al., 2020). In immunocompromised individuals, *P. aeruginosa* is a major pathogen and causes both acute and chronic infections in the respiratory tracts of patients with cystic fibrosis (CF) (Reynolds and Kollef, 2021). Unfortunately, there are limited single-drug or combinatorial treatment options for MDR *P. aeruginosa* infections and physicians frequently resort to antimicrobial chemotherapeutic agents which can be associated with significant side effects (Dean, 2016). To solve this problem, we and others propose developing alternative methods to combat MDR *P. aeruginosa* by using therapeutic monoclonal antibodies (Motley and Fries, 2017; Merakou et al., 2018; Tümmler, 2019).

Administration of antibodies as therapeutics, commonly described as passive immunization, is a method that has been used to treat infectious disease since the late 1800s (Behring and Kitasato, 2013; Graham and Ambrosino, 2015; Kaufmann, 2017). Antibody-based treatments were initially delivered as sera from immune animals containing polyclonal antibodies against the infectious agent (Janeway, 1945; Hammon et al., 1954; Casadevall and Scharff, 1994; Behring and Kitasato, 2013). This strategy was crude and could result in significant side effects (Arturo and Scharff, 1995; Graham and Ambrosino, 2015). Since then, antibody-based treatments have evolved to include the administration of intravenous immunoglobulin (IVIG) from healthy individuals (Lee et al., 1999; Perez et al., 2017) and purified monoclonal antibodies (mAbs). The hybridoma method of generating mAbs by Köhler and Milstein (Köhler and Milstein, 1975) was the first advance into therapeutic mAbs and set the stage for mAbs comprising a significant proportion of pharmaceuticals (Grilo and Mantalaris, 2019). Many therapeutic antibodies are still produced by processes derived from hybridoma technology using mice immunized against a desired antigen (Liu, 2014). With this type of antibody-discovery technology, hundreds of mAbs have been approved for use in humans to treat a variety of conditions, including infectious diseases. To date, most therapeutic infectious disease antibodies approved for human use target viral pathogens such as SARS-CoV-2 (Kreuzberger et al., 2021) and Ebola (Mulangu et al., 2022), but none are approved for use against Gram negative MDR bacterial infections.

Therapies using monoclonal antibodies take advantage of the natural functions of antibodies produced in response to exposure to a pathogen or vaccination. Anti-bacterial antibodies can bind to bacterial cells or neutralize bacterial toxins, stimulate opsonophagocytic killing of the bacterium by phagocytic cells, promote complement deposition, and ultimately facilitate clearance of the pathogen (DiGiandomenico et al., 2014; Hey, 2015; Morrison, 2015; Heesterbeek et al., 2018; Le et al., 2018; Storek et al., 2018; Zurawski and McLendon, 2020). For example, palivizumab (respiratory syncytial virus treatment), bezlotoxumab (*Clostridium difficile* treatment), and bamlanivimab (SARS-CoV2 treatment) are a few of the FDA-approved mAb therapeutics to bacterial and viral pathogens that are used globally and exhibit some of these properties (Beeler and van Wyke Coelingh, 1989; Johnson et al., 1997; Babcock et al., 2006; Johnson and Gerding, 2019). In this work, we hypothesized that anti-*P. aeruginosa* monoclonal antibodies that bind the surface of the bacterium and participate in opsonophagocytic clearance can help with the prevention and treatment of infections caused by this pathogen. This hypothesis is supported by the fact that patients who have convalesced from *P. aeruginosa* infections produce effective polyclonal antibodies against the bacterium (Jacobson et al., 1987; Lee et al., 1999; DiGiandomenico et al., 2012), highlighting the importance of these antibodies in recovery from infection. Subsequent studies in mice have demonstrated that passively immunizing naïve animals with polyclonal antibodies from vaccinated mice or convalescent humans confers significant protection against *P. aeruginosa* challenge (Lee et al., 1999; DiGiandomenico et al., 2012; Sen-Kilic et al., 2021). In addition, our laboratory has also shown that B cells are an important mechanistic correlate of protection against *P. aeruginosa* pneumonia in mice vaccinated with a whole cell *P. aeruginosa* vaccine (Sen-Kilic et al., 2021). To date, various therapeutic monoclonal antibodies against *P. aeruginosa* have been developed and several mAbs tested have exhibited promising pre-clinical results (Milla et al., 2014; Merakou et al., 2018), including efficacy against biofilms (Welsh et al., 1984; Moon et al., 1988; Zaidi et al., 2006; Thompson et al., 2018). Despite several mAb therapeutics evaluated in clinical trials, there is still no approved mAb by the FDA for the treatment of *P. aeruginosa* infections in humans (DiGiandomenico et al., 2012; Patel et al., 2017; Le et al., 2018; Ali et al., 2019; Chastre et al., 2020).

In this study, we generated a novel mAb against *P. aeruginosa* (WVDC-5244) using hybridoma-based technology. ELISA, Western blotting, and flow cytometry were used to characterize WVDC-5244 binding to *P. aeruginosa* and determine the antibody subtype. We demonstrated that WVDC-5244 helps opsonize and kill *P. aeruginosa in vitro* and observed therapeutic activity in a pre-clinical model of acute murine pneumonia. Overall, our study describes a promising mAb candidate for anti-*P. aeruginosa* therapeutic treatment.

## Methods

### Strains of bacteria and growth conditions

For murine challenge experiments, *P. aeruginosa* PAO1 (Dr. Michael L. Vasil, University of Colorado) was used from a frozen stock and was grown on Lysogeny Agar (LA) at 37°C overnight. A single colony from the LA plate was grown in 3 mL of Lysogeny Broth (LB, Miller formulation) overnight and diluted 1:100 in 3 mL of fresh LB and grown over 6 h unless otherwise specified. *P. aeruginosa* clinical isolates from patients with cystic fibrosis(CEC86 CEC32, CEC44, CEC55, CEC60, CF63, CF76, CF154, CF197) were obtained courtesy of Dr. Robert Ernst (University of Maryland: Baltimore) (Burns et al., 2001) other clinical isolates were obtained from WVU Mountain State Cystic Fibrosis Center (**Table S1**). Whole genome sequences of clinical isolates were used to determine strain serotypes using *in silico* serotyping tool Past 1.0 (Thrane et al., 2016). Mutants used for target binding characterization assays were sourced from the PAO1 transposon mutant library and are listed in **Table S1** (Jacobs et al., 2003). Mutants in the gene *mucA* and *wbpM* were kindly provided by Drs. Ohman (Mathee et al., 1999) (VCU) and Goldberg (Emory). *In vivo* passaged mouse isolates were obtained by infecting mice as described below, plating lung homogenates 16 hours post-infection, and isolating individual colonies from distinct mice (M1 and M2). For *in vitro* experiments, *P. aeruginosa* strains were grown on Pseudomonas Isolation Agar (PIA) (Fisher Scientific: BD292710) prior to use unless otherwise specified.

### Vaccination of mice against *Pseudomonas aeruginosa*

Mice were vaccinated with 15 µg of recombinant FpvA protein purified from *Escherichia coli* strain ClearColi (Lucigen) as described previously (Sen-Kilic et al., 2019). The vaccine (200 µL) was administered intraperitoneally and was comprised of equal parts FpvA diluted in Phosphate Buffered Saline (PBS) (100 µL) (Thermo Fisher Scientific: MT21040CV) and Complete Freund’s Adjuvant (100 µL) (*In vivo*gen: vac-cfa-10) to 7-week old BALB/c mice. After 21 days, mice were boosted with 200µL of vaccine comprised of 100 µL (15 µg) of recombinantly produced FpvA adjuvanted with an equal volume (100 µL) of Incomplete Freund’s Adjuvant (IFA) (*In vivo*gen: vac-ifa-10). Four weeks after boost and three days before hybridoma preparation, mice were administered an additional boost vaccine described above.

### Generation of hybridomas from mice

One week prior to fusion, P3X63Ag8.653 myeloma cells (ATCC^®^: CRL-1580™) for hybridomas were thawed and cultured in the ClonaCell-HY Hybridoma Kit (Stem Cell Technologies: 03800) Medium A in 75cm^2^ tissue culture flasks (Greiner Bio-One: 658175). Cells were passaged when 70% confluent until a total number of 10^7^ viable cells were available. At this time, the myeloma cells were combined into a total volume of 15 mL. Mice vaccinated as described above were euthanized with CO_2_ and cervically dislocated. Spleens were removed into 0.5 mL Dulbecco’s Modified Eagle Medium (DMEM) (ThermoFisher Scientific: MT10013CM) with 10% Fetal Bovine Serum (FBS) (Corning Cellgro: 35-010-CV) and homogenized with a pestle. The homogenate was pelleted at 400 x *g* for 10 min at 4°C in a Sorvall Legend Micro 21R centrifuge. The pellet was resuspended in 1 mL DMEM with 10% FBS and filtered through a 100µm cell strainer. Large pieces of tissue were dissociated on the strainer with a plunger and were washed with 4mL DMEM with 10% FBS. The 5 mL cell suspension was pelleted at 400 x *g* for 10 min in a Sorvall RT6000B refrigerated centrifuge and resuspended in 30 mL DMEM without FBS. This process was repeated twice before resuspending cells in 15 mL Medium B from the ClonaCell-HY Hybridoma Kit. Hybridoma fusion was completed per the manufacturer’s instructions of the ClonaCell-HY Hybridoma. Briefly, 2 x 10^8^ myeloma cells were mixed with 10^7^ splenocytes in Medium B. Cells were spun down at 400 x *g* for 10 min at 4°C in a Sorvall RT6000B refrigerated centrifuge. The cell pellet was resuspended by tapping. Polyethylene glycol (PEG) (1 mL) was added dropwise to the cells over one minute without stirring. The cells were then stirred for one minute. Medium B (4 mL) was added drop by drop over four min with stirring. Next, 10 mL of Medium B were added, and the cells were incubated at 37°C for 15 min. Cells were then washed with 30 mL of Medium A and spun down at 400 x *g* for 7 min in a Sorvall RT6000B refrigerated centrifuge. The supernatant was removed, and the cells were washed with 40mL of Medium A. Cells were pelleted at 400 x *g* for seven min in a Sorvall RT6000B refrigerated centrifuge and reconstituted in 10mL of Medium C. The reconstituted cells were transferred to a 75cm^2^ cell culture treated flask and incubated at 37°C with 5% CO_2_ for 24 h.

### Growing, isolating, and screening of hybridoma clones

One day after fusion, cells were prepared for semi-solid plating using the ClonaCell-HY Hybridoma Kit. Hybridomas in Medium C from fusion were centrifuged at 400 x *g* for 10 min in a Sorvall RT6000B refrigerated centrifuge at room temperature in a 50mL conical tube. The supernatant was removed, and the cells were resuspended in 10mL of Medium C. The 10mL of cells were transferred to 90mL of Medium D. The mixture was inverted several times to ensure even distribution of cells throughout the semi-solid medium. Medium was allowed to equilibrate for 15 min at room temperature. After 15 min, 9.5mL of suspension was plated into 100mm x 15mm Petri dishes using a 30mL syringe with a 16-gauge blunt-end needle. Plates were incubated at 37°C with 5% CO_2_ for 10-14 days. Hybridoma colonies were removed from the plates using a 20µL pipette set to 10µL. Colonies were transferred into 200µL of Medium E in 96-well tissue culture treated plates (Greiner Bio-One: 655180). Hybridomas were incubated for 2-3 days at 37°C + 5% CO_2_. Aliquots (100µL) of supernatants were removed every 2 days to test for antigen specificity by ELISA against intact *P. aeruginosa* cells. Briefly, Costar 96 well high-binding microtiter plates (Pierce: 15041) were coated with a volume of 2 × 10^7^ CFU of PAO1 *P. aeruginosa* (50 µL/well) grown on PIA overnight. After coating, the plates were washed three times with 200µL of PBS with 0.05% Tween 20 (PBS-T) (Sigma Aldrich P1379-1L) and blocked with 2% Bovine Serum Albumin (BSA) (Research Products International: 9048-46-8) in PBS overnight at 4°C. Blocked plates were washed three times with PBS-T. Subsequently, 100 µL aliquots of hybridoma cell culture supernatants were transferred to the wells and incubated for 1 h at 37°C. The plates were washed four times with PBS-T and incubated with anti-IgG (Novus: NBP1-75130, 1:2000) horseradish peroxidase conjugated antibodies per well for 1 h at 37°C. Plates were then washed five times with PBS-T and incubated with a 1:1 mixture of TMB substrates A (Biolegend: 77247) and B (Biolegend: 77248) for 30 min following the manufacturer’s instructions. The absorbance of the plates was read at 620 nm using Synergy H1 (BioTek) spectrophotometer. Hybridomas secreting antibodies that bound *P. aeruginosa* were passaged into tissue culture treated 24-well plates (Falcon: 353504) at a ratio of 1:5 in Medium E when confluent in 96-well plates. Once confluent in 24-well plates, hybridomas were transitioned to DMEM + 10% FBS media over the course of 4-6 days. Cells were passaged 1:1 into 50% Medium E with 50% DMEM and 10% FBS on day 0, and into 100% DMEM + 10% FBS on day 4. After this, hybridoma cells were maintained in DMEM + 10% FBS.

### FiberCell production of WVDC-5244

The hybridoma cell line producing WVDC-5244 was grown in Dulbecco’s Modified Eagle Medium (DMEM) (ThermoFisher Scientific: MT10013CM) + 10% v/v Fetal Bovine Serum (FBS) (Corning Cellgro: 35-010-CV) in 75cm^2^ flasks until confluent. WVDC-5244 hybridoma cells (10^8^ cells) were seeded into a medium FiberCell cartridge of high molecular weight cutoff (FiberCell Systems: C2011) per the manufacturers protocol. Cells were weaned off of DMEM + 10% FBS onto chemically defined medium for high density cell culture (CDM-HD) medium (FiberCell Systems: SCD030-0184) and WVDC-5244 was harvested every two-three days for approximately 30 days. Aliquots of WVDC-5244 (0.1mL) were frozen at -80°C until use.

### ELISAs

WVDC-5244 titers against various antigens were assayed by ELISA in Costar 96 well high-binding microtiter plates (Pierce: 15041). The microtiter plates were coated with a volume of 50 µL/well of 2 × 10^7^ CFU of PAO1 or *P. aeruginosa* clinical isolates grown on PIA overnight. After coating, the plates were prepared as described above, =. =. WVDC-5244 mAb was prepared at a concentration of 2 µg/mL in 2% BSA in PBS and serially diluted 1 to 1:64. After 1 hour of incubation at 37°C, the plates were washed four times with PBS-T and incubated with anti- IgG (Novus: NBP1-75130, 1:2000), -IgG1 (Invitrogen: PA174421, 1:5000), -IgG2a (ThermoFisher Scientific: A10685, 1:2000) or -IgG2b (ThermoFisher Scientific: M32407, 1:2000), -IgG3 (ThermoFisher Scientific: M32707, 1:2000) horseradish peroxidase conjugated antibodies at manufacturer recommended dilutions per well for 1 h at 37°C. Plates were then washed five times with PBS-T and incubated with a 1:1 mixture of TMB substrates A (Biolegend: 77247) and B (Biolegend: 77248) for 30 min following the manufacturer’s instructions. A total of 50 µl of 2N H_2_SO_4_ were added to stop the development. The absorbance of the plates was read at 450 nm = using a SpectraMax i3 spectrophotometer (Molecular Devices LLC).Antibody titers were determined as the highest dilution of the sample with a signal exceeding the cut off, calculated as the absorbance of the negative control plus three times the standard deviation of the control. Titer or area under the curve were calculated from the corresponding absorbance values using GraphPad Prism.

### Fluorescence correlation spectroscopy measurements

WVDC-5244 mAb was labeled with Alexa 594 probe (Invitrogen) for FCS experiments. Briefly, the Alexa Fluor 594 reactive dye has a succinimidyl ester moiety that reacts efficiently with primary amines of WVDC-5244 to form stable dye-protein conjugates. The dye labeled WVDC-5244 was purified using a spin column removing the unreacted Alexa 594. Purified Alexa-594 labeled WVDC-5244 were quantified by a UV-visible (UV-vis) spectrometer (Nanodrop 2000). Dye-to-protein ratios were determined to be ~3 by measuring absorbance at 280 nm (protein) versus 594 nm (dye).

*P. aeruginosa* PAO1 and *E. coli* strain ClearColi were grown on LB overnight. An optical density of 0.6 (OD_600_) was used for performing solution binding assay using FCS. The bacterial strains were incubated with 10nM of Alexa Fluor 594-conjugated mAb WVDC-5244 for 90 min at room temperature. FCS measurements were performed in a confocal microscope (ISS Q2) which has single-molecule detection sensitivity. The excitation source was Fianium SC-400 super-continuum laser. NKT super-select AOTF filter was used to select the excitation wavelength of 532 nm which was reflected by a dichroic mirror to a high-numerical-aperture (NA) water objective (60x; NA 1.2) and focused onto the solution sample. The fluorescence was collected by avalanche photodiodes through a dichroic beam splitter and a band-pass (575 – 635 nm; Chroma) filter, thus eliminating the scattered excitation light and collecting the fluorescence from the Alexa Fluor 594 probes in the region of interest. The data acquisition was enabled by a B&H SPC-150 card operated in a photon time-tag time-resolved (TTTR) mode. ISS VistaVision software was used to generate the auto-correlation plots and analyze the FCS data to assess the *in vitro* binding of mAb WVDC-5244 to bacteria. The auto-correlation data of Alexa Fluor 594-conjugated mAb WVDC-5244 was fitted with single species 3D-translational diffusion model suggesting a monomeric population of fluorescently labeled WVDC-5244. Upon reaction of Alexa 594 labeled WVDC-5244 to *P. aeruginosa* PAO1 or *E. coli* strain ClearColi, the autocorrelation plots shifted to longer timescale and the data was fitted with two-species diffusion model. The percentage of total WVDC-5244 that shifts into the more slowly diffusing species, which is the microbe-bound fraction, reflects the relative magnitude of binding in the target population of *P. aeruginosa* PAO1 or *E. coli* strain ClearColi. The FCS measurements and analyses were performed similar to previously reported (Ray et al., 2014; Agrawal et al., 2019).

### Flow cytometry binding assay

The bacteria were resuspended into 1mL of PBS and diluted to 10^8^ CFU/mL. One hundred µL of bacteria (10^7^) were exposed to a range of concentrations of WVDC-5244 or B11 (ThermoFisher-Scientific: MA1-83430) (1.67 x 10^-5^M – 2.6 x 10^-7^M), or medium control. Bacteria-antibody complexes were allowed to form at 4°C for 1 h. Complexed bacteria were removed by centrifugation at 15,000 x *g* for 5 min in a Sorvall Legend Micro 21R centrifuge and washed with 1mL PBS. Bacteria were re-pelleted and exposed to rabbit-anti-mouse 1:400 anti-IgG Alexa-Fluor 488 secondary antibody (ThermoFisher-Scientific: A11059) for 1 h at 4°C in the dark. Stained bacteria were removed by centrifugation at 15,000 x *g* for 5 min in a Sorvall Legend Micro 21R centrifuge and washed with 1 mL PBS. Cells were then fixed for 1 h at 4°C with 4% v/v paraformaldehyde (Sigma-Aldrich: P6148-500G) or overnight at 4°C with 0.4% paraformaldehyde. Post-fixation, cells were centrifuged at 15,000 x *g* for 15 min in a Sorvall Legend Micro 21R centrifuge and reconstituted in 300µL of PBS. Samples were analyzed by flow cytometry on a BD Fortessa. Bacteria were gated on low FSC SSC profile and +/- Alexa-Fluor 488 intensity.

### J774A.1 macrophage cell culture conditions

J774A.1 (ATCC^®^: TIB-67) macrophages (Ralph and Nakoinz, 1975) were cultivated in 20mL of DMEM with 10% v/v FBS in 75cm^2^ tissue culture treated flasks (Greiner Bio-One: 658170) at 37°C with 5% CO_2_. Cells were grown to ~70% confluence before passaging. Confluent macrophages were removed from flasks with a cell scraper (VWR: 101093-454) and centrifuged at 300 x *g* for 10 min in a Sorvall RT6000B refrigerated centrifuge. Cells were reconstituted in 20mL of fresh DMEM with 10% v/v FBS.

### Opsonophagocytic killing assays

Opsonophagocytosis was assessed by an assay modified from previous studies (Ames et al., 1985; Pier et al., 1987; Sen-Kilic et al., 2019). *P. aeruginosa* PAO1 was grown on PIA overnight at 37°C. Single colonies were selected and inoculated into 3mL of Minimal M9 Broth with 0.2% glucose (M9) (Teknova B8003) overnight. The following day, the suspensions were diluted 6-fold in fresh M9 and grown over 6 h. The cells were pelleted and re-suspended in 5mL of Eagle’s Minimum Essential Medium (Corning Cellgro: 10-009-CV) + 1% w/v bovine serum albumin (MEM-BSA). The suspensions were diluted in MEM-BSA to obtain a final concentration of 2 x 10^7^ CFU/mL. J774A.1 macrophages were harvested by centrifugation at 400 x *g* for 10 min in a Sorvall RT6000B refrigerated centrifuge and resuspended in MEM-BSA at a concentration of 10^6^ cells/mL. WVDC-5244 (75 µg) or negative control (-Ab: hybridoma medium) was added to the bacteria and macrophages. Bacteria, macrophages, and WVDC-5244 were incubated together in microcentrifuge tubes by tumbling end-over-end on a rotator at 37°C for 2 h. At the beginning and end of the incubation period, samples were diluted 1:9 in water to lyse the macrophages, and then serially diluted (ten-fold) in PBS and plated on PIA. The percentage of bacterial killing was calculated by comparing the number of bacteria killed in samples incubated with WVDC-5244 to the negative control.

### Complement killing assay

To determine the ability of WVDC-5244 to mediate complement killing, 10^6^ CFU/mL *P. aeruginosa* PAO1 were grown according to the standard procedure and opsonized with 30μg/ml of WVDC-5244 and 30% v/v of Guinea Pig Complement (MP Biomedicals TM Guinea Pig Complement, #ICN642831, Fisher Scientific) in a final volume of 100 μL. Samples were taken at time 0 and 90 min. To determine the bacterial viability, samples were diluted in PBS, plated on PIA and incubated at 37°C overnight. The percentage of bacterial survival was calculated relative to the number of bacteria at 0 min.

### *In vivo* infections and WVDC-5244 administration

Therapeutic efficacy of WVDC-5244 was evaluated in a prophylaxis model of murine pneumonia. Six-week-old female inbred BALB/c mice received a dose of 15 mg/kg of WVDC-5244 in PBS or PBS alone as control (n=10). After 9 h, 20µL (10^7^ CFU) of *P. aeruginosa* PAO1 grown as described above was administered intranasally to mice anesthetized with ketamine (77 mg/kg) (Patterson Veterinary: 07-803-6637)/xylazine (7.7 mg/kg) (Patterson Veterinary: 07-808-1794). The endpoint for these challenge experiments was determined as 16 h to minimize animal distress. Two independent experiments with 5 mice per group were conducted and the data were combined.

After 16 h, the animals were euthanized with 390 mg/kg of pentobarbital in 0.9% NaCl (Patterson Veterinary: 07-805-9296). The lung wet weights were measured. Lung homogenates were generated by dissociating the lungs with a Brinkmann PT-10-35 Polytron Homogenizer and nasal washes were collected by rinsing the nasal cavity with 1 mL of PBS. One hundred µL of lung homogenates or nasal washes were serially diluted (ten-fold) in sterile PBS. Diluted homogenates were plated on PIA to determine viable *P. aeruginosa* CFUs.

### Statistical analyses

Statistical differences between experimental groups were assessed using appropriate statistical tests. Differences between multiple groups were assessed with a one-way ANOVA followed by Tukey’s multiple comparisons test for normally distributed data and Kruskal Wallis Dunn’s Multiple Comparison’s test was performed for nonparametric data. Differences between two groups were identified using two-tailed student’s *t*-tests (parametric) or Mann-Whitney test (non-parametric). Significant differences were defined as groups meeting the requirement *P* < 0.05. Graphpad Prism version 9.2.0 was used for all statistical analyses performed. All of the *in vitro* experiments performed in at least in technical and/or biological duplicates.

## Results

### Generation of mAbs against *Pseudomonas aeruginosa*

**Figure 1:**
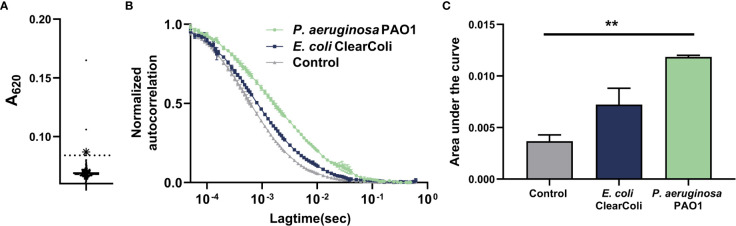
Generation of a monoclonal antibody that opsonizes *P. aeruginosa*. **(A)** ELISA of antibodies binding *P. aeruginosa* generated by hybridomas produced from vaccinated mice. WVDC-5244 is highlighted in blue. **(B)** Single molecule solution-based assay by FCS to determine the binding of fluorescently labeled WVDC-5244 to *P. aeruginosa* PAO1 and (*E*) *coli* strain Clear-coli. **(C)** Area under the curve (AUC) representation of **(B)**. One-way ANOVA was used for statistical analysis. The asterisk refers to statistical significance: **p ≤ 0.01. Error bars indicate SDs.

Previous work from our laboratory has focused on the development of sub-unit vaccines against *P. aeruginosa (* Sen-Kilic et al., 2019). From this work, we hypothesized that the antigens used in our studies, such as the ferripyoverdine receptor FpvA, could be used to generate mAbs against *P. aeruginosa*. Mice were vaccinated against purified *P. aeruginosa* FpvA, and splenocytes from vaccinated mice were extracted and fused with myeloma cells. Hybridoma fusion and selection resulted in 29% of recovered hybridoma colonies producing stable cell lines that were subsequently grown in cell-culture plates for determination of antigen specificity (data not shown). Once cultured hybridomas reached confluence in 96-well plates, we used a direct ELISA assay to select for hybridomas producing IgG binding to the surface of intact *P. aeruginosa* PAO1 cells. Of the total hybridomas, 2% produced IgG against *P. aeruginosa* PAO1 above detection threshold (**Figure 1A**). Among these antibodies, we identified an antibody, hereby called WVDC-5244, capable of interacting with *P. aeruginosa* (**Figures 1B, C**). Since the recombinant protein used as a vaccine antigen was expressed in *E. coli*, we also determined WVDC-5244 binding to the strain of *E. coli* that was used for recombinant antigen production. We observed that WVDC-5244 did not significantly bind to *E. coli* compared to the PBS control (**Figures 1B, C**). After initial screening, WVDC-5244 was selected for subsequent characterization studies and production was scaled up in a hollow-fiber bioreactor.

### WVDC-5244 binds to clinical strains of *Pseudomonas aeruginosa* from different LPS serotypes and with varying levels of mucoidy

*P. aeruginosa* isolates that cause clinical *P. aeruginosa* infections are often phenotypically and genotypically different from lab-adapted strains (Shen et al., 2006; Huse et al., 2010; Grosso-Becerra et al., 2014; Chandler et al., 2018; Subedi et al., 2018). For example, *P. aeruginosa* strains have been classified into 20 different LPS serotypes based on the structure of their O-polysaccharide (Raymond et al., 2002; Lam et al., 2011), and chronic isolates of *P. aeruginosa* can lose O-polysaccharide expression (Burns et al., 2001; Dettman et al., 2013). Further differences between lab-adapted, acute clinical isolates, and chronic clinical isolates include expression of the exopolysaccharide alginate (characteristic of chronic isolates, mediates resistance to stress), antibiotic resistance, motility, and PQS expression [a molecule involved in the production of OMVs (Schertzer and Whiteley, 2012; Horspool and Schertzer, 2018) that can confer antibiotic resistance (Kadurugamuwa and Beveridge, 1997; Ho et al., 2015; Jan, 2017)] among others.

**Figure 2:**
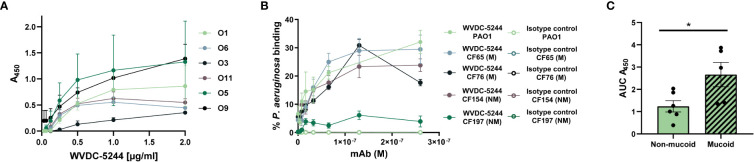
WVDC-5244 binds to clinical *P. aeruginosa* isolates from varying LPS serotypes and mucoidy. **(A)** ELISA curves of WVDC-5244 binding to *P. aeruginosa* clinical isolates. **(B)** Percentage of binding of *P. aeruginosa* PAO1, and mucoid and non-mucoid *P. aeruginosa* clinical isolates incubated with IgG2b isotype control or WVDC-5244. **(C)** AUC analysis of WVDC-5244 binding to mucoid and non-mucoid *P. aeruginosa* clinical isolates. Unpaired two-tailed *t*-test was used for statistical analysis. The asterisk refers to statistical significance: *p ≤ 0.05. Error bars indicate SEMs.

As we strive to generate immunotherapies against clinically relevant *P. aeruginosa* strains, we investigated whether WDC-5244 was capable of binding and opsonizing clinical isolates of *P. aeruginosa*. To do this, we selected phenotypically and genotypically diverse MDR *P. aeruginosa* strains isolated from pediatric patients with cystic fibrosis (**Table S1**). The strains selected expressed different LPS serotypes, and exhibited phenotypic differences in mucoidy, colony shape, color and size, motility, and PQS expression. When comparing binding across serotypes using ELISA, we observed that WVDC-5244 was capable of binding to clinical isolates from all the LPS serotypes tested (O1, O3, O5, O6, O9, and O11) (**Figures 2A**, **S1**). In addition, flow cytometry binding assays demonstrated that WVDC-5244 was able to bind to both non-mucoid and mucoid clinical strains of *P. aeruginosa* (**Figures 2B, C**). Unexpectedly, we observed that binding was significantly higher to mucoid compared to non-mucoid strains (**Figure 2C**). Interestingly, we observed using this assay that only a maximum of 30% of the bacteria are opsonized by WVDC-5244. To determine if population staining heterogeneity was due to assay limitations or heterogenous expression of the antigen target, we performed the assay with the B11 antibody that recognizes a *P. aeruginosa* outer-membrane protein. Interestingly, we observed that B11 was only able to bind approximately 20% of 2 of the clinical isolates tested (**Figure S2**). This suggests that the relatively low (20-30%) maximum opsonization observed for both antibodies is potentially an experimental artifact, although it does not exclude that the targets of WVDC-5244 and B11 might also be heterogeneously expressed on the surface of *P. aeruginosa*. Overall, the data demonstrate that WVDC-5244 can bind and opsonize not only laboratory strain PAO1 but also clinical strains of *P. aeruginosa*, regardless of their LPS serotype and mucoidy.

### WVDC-5244 is an IgG2b that mediates complement opsonization and opsonophagocytic killing of *Pseudomonas aeruginosa*

**Figure 3:**
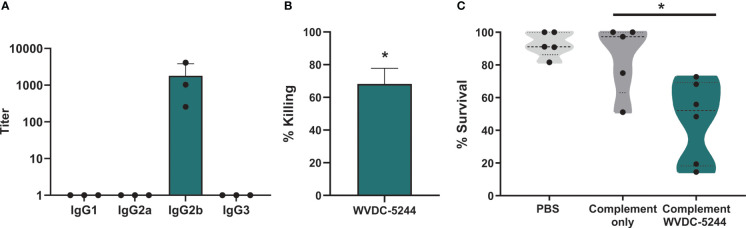
WVDC-5244 is an IgG2b that mediates killing of *P. aeruginosa*. **(A)** ELISA of WVDC-5244 against PAO1 using secondary antibodies to murine IgG1, IgG2a, IgG2b, or IgG3 **(B)** Percentage of opsonophagocytic killing of *P. aeruginosa* in the WVDC-5244 treated group compared to PBS control. **(C)** Percent of *P. aeruginosa* survival in presence of Guinea pig complement with or without WVDC-5244. The *p*-values calculated using Kruskal Wallis Dunn’s Multiple Comparison’s test. The asterisk refers to statistical significance: *p ≤ 0.05. Error bars indicate SDs.

The F_c_ region of antibodies plays an essential role in their function against pathogens (Mancardi et al., 2008; Bruhns, 2012; Lu et al., 2018; Saunders, 2019). Particularly in the context of infectious disease, differences in antibody subtypes can result in changes to the immunological response (Lu et al., 2018). We performed ELISA to determine the sub-class of the antibody. We identified that WVDC-5244 is an IgG2b mAb (**Figure 3A**). IgG2b are a type of antibody classically associated with opsonization of antigens with a high epitope density (Aase and Michaelsen, 1994). They are involved in the clearance of pathogens (Hektoen, 1909; Reynolds et al., 1975; Joller et al., 2010; Lu et al., 2018) primarily *via* FcγR activation of phagocytic leukocytes (Mancardi et al., 2008; Bruhns, 2012). To characterize the effector functions of WVDC-5244, we first performed opsonophagocytic killing assays. *P. aeruginosa* PAO1 was incubated in the presence of WVDC-5244 and J774A.1 macrophages and survival of *P. aeruginosa* was quantified after 2 hours. We observed that incubation of *P. aeruginosa* with WVDC-5244 and macrophages led to the killing of 68.2% of bacteria compared to macrophages alone (**Figure 3B**). In addition to opsonophagocytosis, we also performed complement-mediated killing assays. PAO1 was incubated with either PBS as control, complement alone, or WVDC-5244 and complement. We observed that complement alone was insufficient to significantly reduce bacterial viability (**Figure 3C**). However, combination of complement with WVDC-5244 led to a 53.5% decrease in bacterial survival (**Figure 3C**). Overall, these results show that WVDC-5244 is able to mediate opsonophagocytic and complement-mediated killing of *P. aeruginosa.*

### WVDC-5244 prophylactic treatment reduces bacterial load in the respiratory tract during murine *Pseudomonas aeruginosa* pneumonia

**Figure 4:**
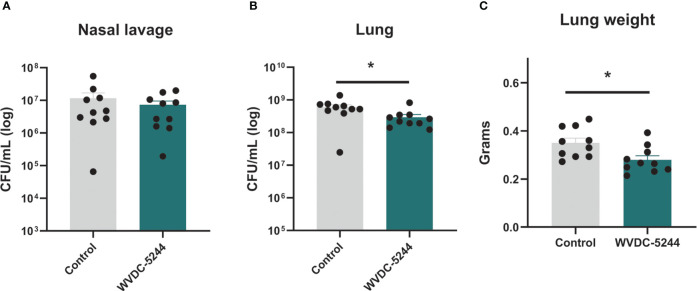
WVDC-5244 treatment reduces bacterial burden during *P. aeruginosa* acute murine pneumonia. *P. aeruginosa* burden in the nasal lavage **(A)** or lung **(B)** and lung weight **(C)** 16 hrs post-infection were quantified in a BALB/c acute murine model of infection. BALB/c mice received 0.375 mg of total WVDC-5244 antibody 9 hrs prior to challenge with 10^7^ CFU of *P. aeruginosa* PAO1. Control groups received equivalent amount of PBS. Each dot represents single mouse. Statistical analyses were performed using Mann-Whitney test. The asterisk refers to statistical significance: *p ≤ 0.05. Error bars indicate SEMs.

We next evaluated WVDC-5244 in a murine challenge model. Respiratory *P. aeruginosa* infections are notoriously difficult to treat and are severe for immunocompromised individuals (Deretic et al., 1995; Tümmler and Kiewitz, 1999; Horrevorts et al., 2012; Bhagirath et al., 2016; Malhotra et al., 2019). In murine models of acute pneumonia, *P. aeruginosa* infections typically result in lung edema and bacterial colonization of the nares and lungs (Sadikot et al., 2005; Sawa, 2014; Sen-Kilic et al., 2019). To determine the therapeutic potential of WVDC-5244, we performed passive immunization in BALB/c mice. Nine hours after injection, mice were challenged intranasally with PAO1. Sixteen hours post-challenge, wet lung weight and respiratory bacterial colonization were evaluated. While administration of WVDC-5244 had a limited effect on bacterial burden in the nares (37% reduction in BALB/c mice, **Figure 4A**), we observed that WVDC-5244 treatment significantly reduced *P. aeruginosa* infection in the lungs by 51% (**Figure 4B**). WVDC-5244 treatment also significantly reduced lung wet weight in BALB/c mice which is an indirect measurement of inflammation and edema associated with infection (**Figure 4C**). Overall, our data show that WVDC-5244 can modestly but significantly reduce bacterial burden in the lower respiratory tract when administered prophylactically during *P. aeruginosa* acute murine pneumonia.

In summary, we generated a monoclonal antibody that can bind and opsonize *P. aeruginosa* PAO1 and clinical isolates of this bacterium. We showed that WVDC-5244 monoclonal antibody can facilitate opsonophagocytic and complement mediated killing *in vitro*. In an acute murine pneumonia model, we demonstrated that prophylactic treatment of mice with WVDC leads to a reduction in lung bacterial burden in both inbred and outbred strains. Overall, this study provides valuable pre-clinical efficacy data for the use of WDC-5244 in the prevention of *P. aeruginosa* infection.

## Discussion

Antibody therapeutics are one of the newest classes of drugs approved for human use. Over the course of the last two decades, there has been a surge in the development and implementation of monoclonal antibodies to treat a variety of diseases, from autoimmune disorders to infectious diseases. Monoclonal antibody therapeutics against *P. aeruginosa* are being developed as an alternative or supplement for conventional antibiotics, as they can provide new answers for the treatment of antibiotic-resistant infections.

In this study, we showed that WVDC-5244 opsonizes both lab-adapted and clinical strains of *P. aeruginosa* (**Figures 1**, **2**). We observed binding of WVDC-5244 to MDR clinical isolates of *P. aeruginosa* expressing various clinically relevant LPS serotypes, and varying mucoidy levels, suggesting its potential utility in clinical applications. While we measured detectable binding to all the isolates tested, there were noticeable differences in antibody binding between each strain. It is likely that these differences are associated with changes in antigen expression, surface availability, or sequence due to the extensive variability observed in clinical *P. aeruginosa* isolates (Shen et al., 2006; Huse et al., 2010; Grosso-Becerra et al., 2014; Chandler et al., 2018; Subedi et al., 2018). In this study, we aimed to generate an antibody that binds to the iron-acquisition receptor FpvA of *P. aeruginosa*. Using Western blotting, our preliminary characterization work shows that WVDC-5244 does not bind to recombinant FpvA (data not shown), but binds to another protein of *P. aeruginosa* (**Figure S3**). This is likely due to the strategy used for antibody screening. Antibodies that bind to the surface of intact cells were selected using ELISA against intact cells to prioritize the identification of antibodies that bind to surface proteins. Future studies focusing on the generation of anti-FpvA antibodies should consider using recombinant FpvA for screening as an additional step to avoid the selection of off-target antibodies. To gain additional insights into the potential target of WVDC-5244, we also evaluated binding to mutants of *P. aeruginosa* lacking expression of various exopolysaccharides including pel, psl, and alginate, or the core of the LPS, or overexpressing alginate. Mutations in the loci tested did not alter binding of WVDC-5244 to *P. aeruginosa*, suggesting that the antibody does not bind the most abundant extracellular polysaccharides expressed by *P. aeruginosa* (**Figures S4A, B**). In addition, *in vivo* passage of *P. aeruginosa* did not affect antibody binding either (**Figure S4C**). Despite extensive attempts at determining the identity of this protein, the binding target of WVDC-5244 remains unknown. Identification of the epitope bound by WVDC-5244 will enable future studies characterizing the levels of expression, conservation, and surface availability across clinical *P. aeruginosa* isolates.

Antibody function is closely related to antibody class and sub-class. WVDC-5244 is an IgG2b mAb, a class of antibody that possesses a range of unique properties relative to antibodies with other F_c_ types. IgG2b antibodies have excellent antimicrobial properties (Oi et al., 1984; Michaelsen et al., 2004) and their constant region facilitates binding to the murine F_c_ receptors FcγRIV, FcγRI, and FcγRIII, and the inhibitory murine F_c_ receptor FcγRIIB (Mancardi et al., 2008; Bruhns, 2012). Downstream signaling facilitated by these receptors is critical in mediating the activity of phagocytic cells. FcγRIV in particular is important for activation of macrophages, monocytes, and neutrophils which have been demonstrated to be important for clearance of *P. aeruginosa* infections (Koh et al., 2009; Lavoie et al., 2011) and is selective for IgG2 subclasses. WVDC-5244 induced opsonophagocytic killing of *P. aeruginosa* by macrophages suggesting that presence of this receptor on phagocytes may be critically important for its efficacy. Future research efforts could focus on modifying the Fc region of WVDC-5244 to alter the characteristics of the antibody and increase its efficacy.

To determine if the *in vitro* complement- and opsonophagocytic-mediated activities of WVDC-5244 translate to *in vivo* efficacy, we tested this antibody in a prophylactic model of acute pneumonia in both inbred mice. We demonstrated that a single dose of WVDC-5244 was sufficient to reduce *P. aeruginosa* bacterial burden at 16 hours post-infection. It is encouraging that these results are comparable to other candidate therapeutic monoclonal antibodies that increase survival or reduce infection in animal models (DiGiandomenico et al., 2014; Le et al., 2018). In this study, we were only able to evaluate the prophylactic use of WVDC-5244 against *P. aeruginosa* pneumoniae and could not evaluate its therapeutic properties. This is due in part to the technical limitations of the acute *P. aeruginosa* pneumonia model in mice and to the narrow therapeutic window for antibody administration. As we continue to evaluate the therapeutic potential of WVDC-5244 as an anti-*P. aeruginosa* therapeutic, inclusion of challenge models with therapeutic interventions–as well as additional models, such as sepsis, wound, keratitis, and chronic lung infections–will be beneficial.

Overall, the data presented here describe a novel anti-*P. aeruginosa* mAb (WVDC-5244). We have demonstrated that this antibody binds *P. aeruginosa*, opsonizes it, mediates opsonophagocytic killing of the bacterium, and reduces *P. aeruginosa* acute pneumonia in mice. While these results are encouraging, future efforts will focus on humanization, improvement of WVDC-5244 *in vivo* efficacy and effector function, and evaluation in combination with current standard of care antibiotics in various pre-clinical models of *P. aeruginosa* infection.

## Data availability statement

The original contributions presented in the study are included in the article/**Supplementary Material**. Further inquiries can be directed to the corresponding author.

## Ethics statement

The animal study was reviewed and approved by West Virginia University Institutional Animal Care and Use Committee protocol # 1606003173.

## Author contributions

AMH, FHD, and MB designed the study. AMH and ES-K prepared and administered the FpvA vaccine. AMH and SLB generated and maintained hybridomas. AMH produced WVDC-5244 in the hollow-fiber bioreactor. AMH, ACM, MAN, SE performed ELISAs to characterize the subtype of WVDC-5244 and assess opsonophagocytosis killing assays. KR, GAS, and GKL performed spectroscopy analyses. AMH, ES-K, MMB, MSH, SJM, JK, CBB, KLW, WTW, ABH and GMP performed animal experiments. TC and SAQ collected clinical isolates of *P. aeruginosa* and provided information on strain origin. All authors assisted with data generation in *in vitro* and *in vivo* experiments. AMH, ES-K and MB wrote the manuscript, and all authors took part in the editing and revision process. All authors contributed to the article and approved the submitted version.

## Supplementary material

Supplementary Figure 1 Detection of WVDC-5244. Representative plots from flow cytometry detection of WVDC-5244 binding to *P. aeruginosa* using a FITC labeled secondary antibody for detection. Control mAb refers to a sample incubated with the secondary antibody alone (no WVDC-5244).

Supplementary Figure 2 Binding of B11 to clinical *P. aeruginosa* isolates. ELISA curves of B11 binding to PAO1 and various clinical isolates with either mucoid (M) or non-mucoid (NM) phenotypes.

Supplementary Figure 3 WVDC-5244 binds *P. aeruginosa* proteins but not LPS. **(A)** Western blot analysis of WVDC-5244 binding to 10 µg of *P. aeruginosa* bacterial lysate. **(B)** Whole-cell lysates of *P. aeruginosa* were treated with proteinase K (PK) (1.3 mg/ml final concentration), with sodium periodate (NaIO_4_) (13.3 nM final concentration) or non-treated, separated on SDS-PAGE gels and immunoblotted with WVDC-5244 mAb 5 µg/ml. **(C)** Western blot using serum from mice previously vaccinated with heat-inactivated whole cell *P. aeruginosa* PAO1 (*Pa-*WCV) or 5 µg/ml of WVDC-5244 against LPS isolated from *P. aeruginosa* PAO1.

Supplementary Figure 4 WVDC-5244 can bind to *in vivo* passaged *P. aeruginosa* PAO1. ELISA curves of WVDC-5244 binding to *P. aeruginosa* PAO1 isolated from the lung of mice 16 hours post-challenge. Data from isolates proceeding from two distinct mice are provided (M1 and M2).
