# Supplementary figures and images for: Development of an anti-Pseudomonas aeruginosa therapeutic monoclonal antibody WVDC-5244
Source: Front Cell Infect Microbiol. 2023 Apr 14;13:1117844. doi: 10.3389/fcimb.2023.1117844 (PMC10140502; doi:10.3389/fcimb.2023.1117844)

**Figure S1**

**
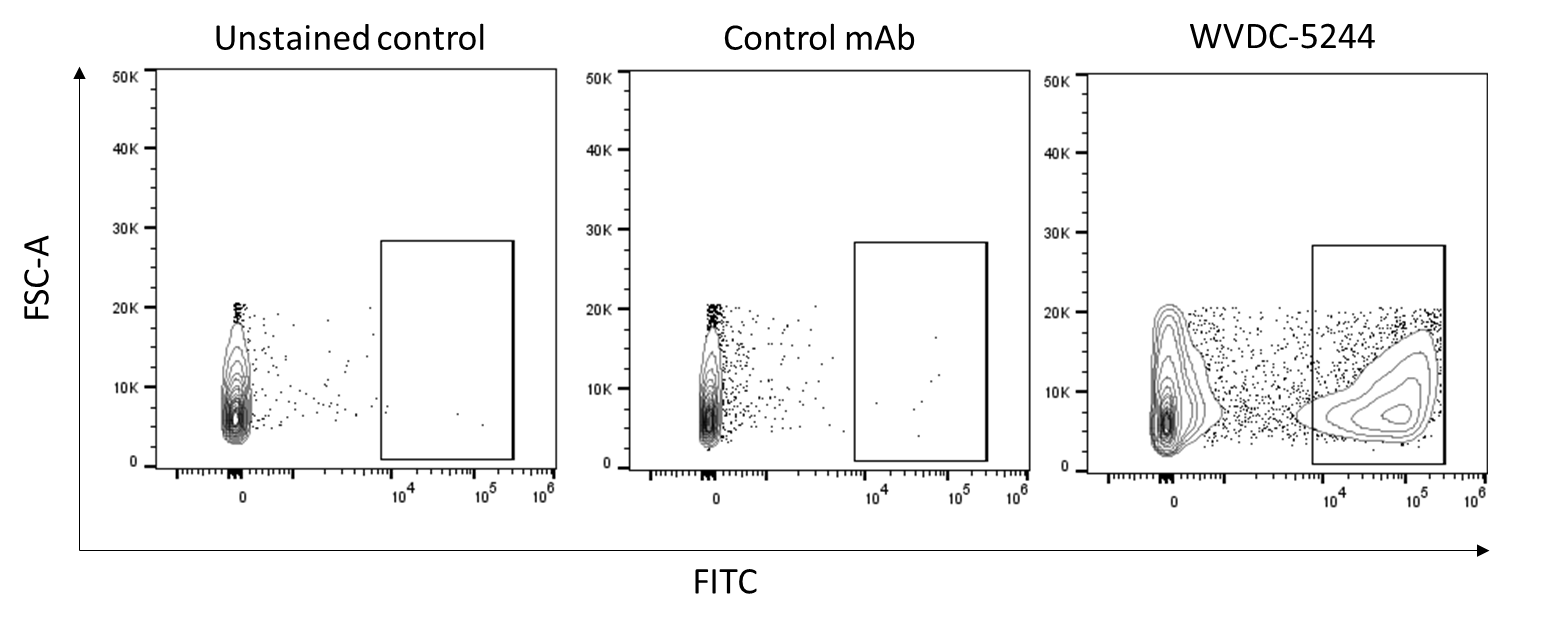
**

**Figure S2**

**
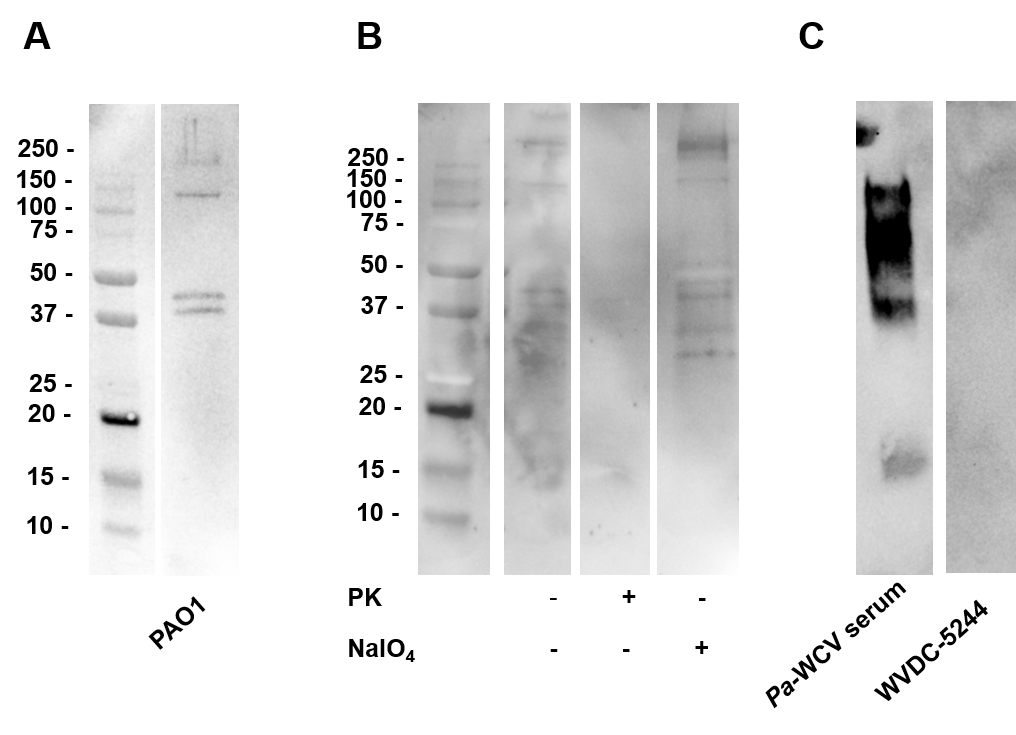
Figure S3**

**Figure S4**


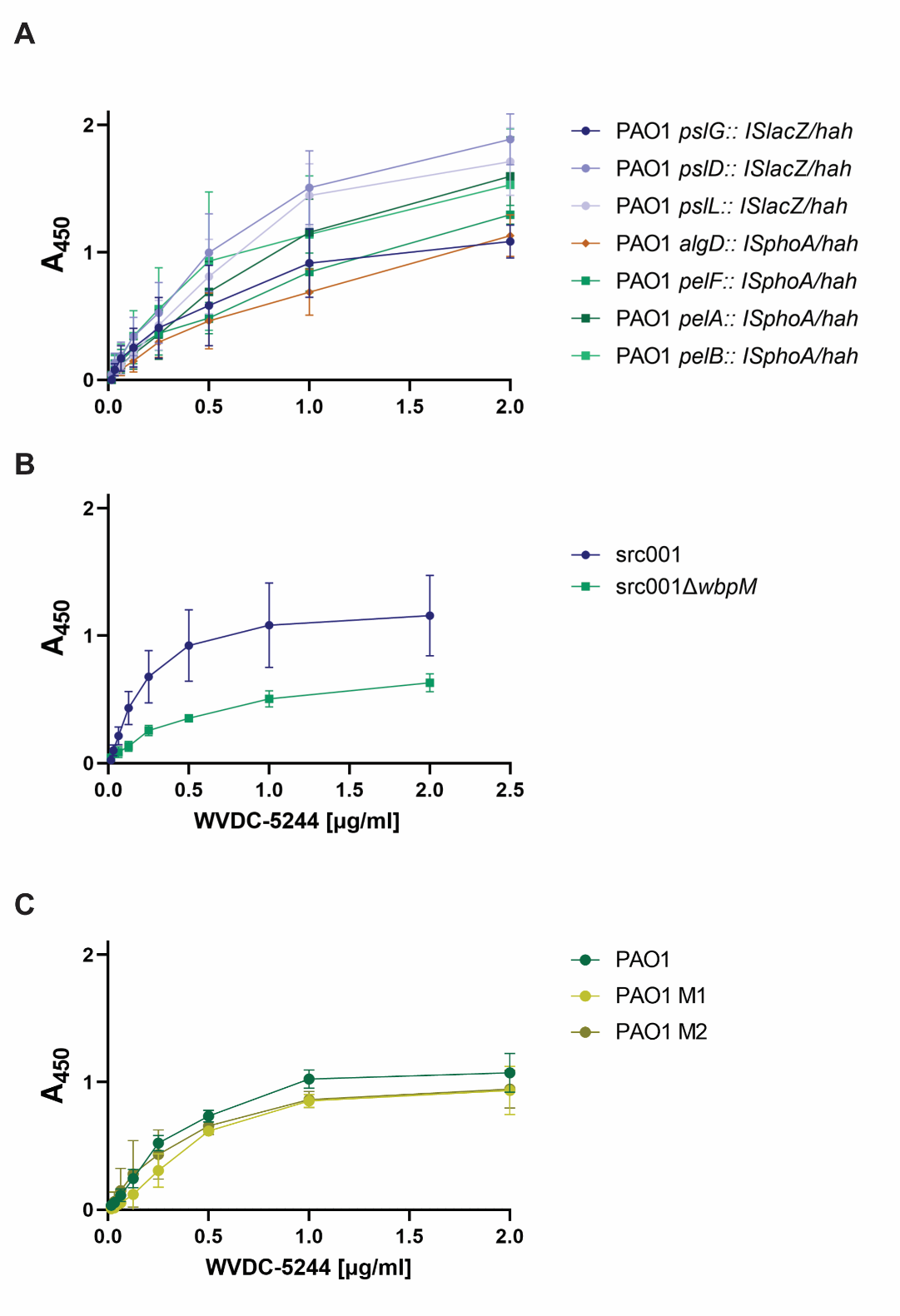


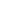


**
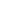

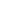
**

Supplement: Supplementary Figure 1 — Detection of WVDC-5244. Representative plots from flow cytometry detection of WVDC-5244 binding to P. aeruginosa using a FITC labeled secondary antibody for detection. Control mAb refers to a sample incubated with the secondary antibody alone (no WVDC-5244). [file DataSheet_1.docx]
