# Supplementary material for: Development of an anti-Pseudomonas aeruginosa therapeutic monoclonal antibody WVDC-5244
Source: Front Cell Infect Microbiol. 2023 Apr 14;13:1117844. doi: 10.3389/fcimb.2023.1117844 (PMC10140502; doi:10.3389/fcimb.2023.1117844)
Supplement: Supplementary file 2 [file Table_1.docx]

**Supplementary Table 1. Bacterial strains used in this study.**

| **Bacterial Strains** | **Source** | **Origin or mutant number** | **Colony Size** | **Colony morphology** | **Mucoidy** | **Serotype** |
| --- | --- | --- | --- | --- | --- | --- |
| *P. aeruginosa* PAO1 | M. Vasil | M. Vasil | M | R | NM | O5 |
| *P. aeruginosa* CEC31 | Burns, et al. 2001 | Patient 3 | M | R | NM | O9 |
| *P. aeruginosa* CEC32 | Burns, et al. 2001 | Patient 3 | S | R | NM | O5 |
| *P. aeruginosa* CEC44 | Burns, et al. 2001 | Patient 7 | M | I | NM | O1 |
| *P. aeruginosa* CEC55 | Burns, et al. 2001 | Patient 4 | M | I | NM | O11 |
| *P. aeruginosa* CEC60 | Burns, et al. 2001 | Patient 104 | S | R | NM | O6 |
| *P. aeruginosa* CEC86 | Burns, et al. 2001 | Patient 108 | S | R | NM | O3 |
| *P. aeruginosa* CF65 (CEC95) | Burns, et al. 2001 | Patient 9 | L | R | M | O6 |
| *P. aeruginosa* CF76 (CEC105) | Burns, et al. 2001 | Patient 9 | M | R | M | O1 |
| *P. aeruginosa* CF153 | Burns, et al. 2001 | Patient 204 | M | R | M | O3 |
| *P. aeruginosa* CF154 | Burns, et al. 2001 | Patient 204 | S | I | NM | - |
| *P. aeruginosa* CF197 | Burns, et al. 2001 | Patient 212 | S | R | NM | - |
| *P. aeruginosa* CF165 (CEC70) | Burns, et al. 2001 | Patient 205 | M | R | M | O6 |
| *P. aeruginosa* CPAP16 | This study | Patient 16 | L | R | M | - |
| *P. aeruginosa* CPAP36B1 | This study | Patient 36 | M | R | M | - |
| *P. aeruginosa* CPAP38B1 | This study | Patient 38 | L | R | M | - |
| *P. aeruginosa* CPAP51 | This study | Patient 51 | M | R | M | - |
| *P. aeruginosa* PAO1 *pslG::* *ISlacZ/hah* | Jacobs, et al. 2003 | PW4808 | M | R | NM | O5 |
| *P. aeruginosa* PAO1 *pslD::* *ISlacZ/hah* | Jacobs, et al. 2003 | PW4802 | M | R | NM | O5 |
| *P. aeruginosa* PAO1 *pslL::* *ISlacZ/hah* | Jacobs, et al. 2003 | PW4812 | M | R | NM | O5 |
| *P. aeruginosa* PAO1 *algD:: ISphoA/hah* | Jacobs, et al. 2003 | PW6998 | M | R | NM | O5 |
| *P. aeruginosa* PAO1 *pelF:: ISphoA/hah* | Jacobs, et al. 2003 | PW6130 | M | R | NM | O5 |
| *P. aeruginosa* PAO1 *pelA:: ISphoA/hah* | Jacobs, et al. 2003 | PW6141 | M | R | NM | O5 |
| *P. aeruginosa* PAO1 *pelB:: ISphoA/hah* | Jacobs, et al. 2003 | PW6138 | M | R | NM | O5 |
| src001 | Goldberg lab |  | M | R | NM | O1 |
| src001Δ*wbpM* | Goldberg lab |  | M | R | NM | O1 |
| PAO1Δ*mucA* | Mathee, et al. 1999 |  | M | R | M | O5 |
| M1 | This study | PAO1 *in vivo* passaged | M | R | NM | O5 |
| M2 | This study | PAO1 *in vivo* passaged | M | R | NM | O5 |

Colony size: S=Small, M=Medium, L=Large

Colony morphology: R=Regular, I=Irregular

Mucoidy: NM=Non-mucoid, M=Mucoid
